# Supplementary material for: Navigating the garden of forking paths for data exclusions in fear conditioning research
Source: eLife. 2019 Dec 16;8:e52465. doi: 10.7554/eLife.52465 (PMC6989118; doi:10.7554/eLife.52465)
Supplement: Transparent reporting form [file elife-52465-transrepform.docx]

***eLife’s* transparent reporting form**

We encourage authors to provide detailed information *within their submission* to facilitate the interpretation and replication of experiments. Authors can upload supporting documentation to indicate the use of appropriate reporting guidelines for health-related research (see [EQUATOR Network](http://www.equator-network.org/%20)), life science research (see the [BioSharing Information Resource](https://biosharing.org/)), or the [ARRIVE guidelines](http://www.plosbiology.org/article/info:doi/10.1371/journal.pbio.1000412) for reporting work involving animal research. Where applicable, authors should refer to any relevant reporting standards documents in this form.

If you have any questions, please consult our Journal Policies and/or contact us: [editorial@elifesciences.org](mailto:editorial@elifesciences.org).

**Sample-size estimation**

- You should state whether an appropriate sample size was computed when the study was being designed
- You should state the statistical method of sample size computation and any required assumptions
- If no explicit power analysis was used, you should describe how you decided what sample (replicate) size (number) to use

Please outline where this information can be found within the submission (e.g., sections or figure legends), or explain why this information doesn’t apply to your submission:

Not applicable, re-analysis of existing datasets for the purpose of case examples

case examples based on re-analysis of two existing data sets

Dataset 1: N = 119 participants, female = 79, male = 40, ageM = 25.0, ageSD = 3.64

Dataset 2: N = 268 participants, female = 195, male = 73, ageM = 25, age SD = 4

**Replicates**

- You should report how often each experiment was performed
- You should include a definition of biological versus technical replication
- The data obtained should be provided and sufficient information should be provided to indicate the number of independent biological and/or technical replicates
- If you encountered any outliers, you should describe how these were handled
- Criteria for exclusion/inclusion of data should be clearly stated
- High-throughput sequence data should be uploaded before submission, with a private link for reviewers provided (these are available from both GEO and ArrayExpress)

Please outline where this information can be found within the submission (e.g., sections or figure legends), or explain why this information doesn’t apply to your submission:

Not applicable, the article is about treating outliers/non-learner

Dataset 1: Since data exclusion is the topic of the paper, n = 3 participants of the overall sample N = 119 were classified as SCR non-responder and excluded from some of the analyses. Non-responder were defined as individuals with more than two thirds of non-responses to the US (more than 9 out of 14 trials).

Dataset 2: Out of the full sample (N = 357), 31 were excluded prior to phyisological processing either due to abortion of the experiment or due to technical failures during data acquisition (e.g. errors during saving, overwritten logfile, or missing markers). 39 participants were excluded because of incomplete questionnaire data (STAI-T). Physiological non-responders were defined as individuals with more than two thirds of non-responses to the US (more than 6 out of 9 trials) and were also excluded (N = 19).

**Statistical reporting**

- Statistical analysis methods should be described and justified
- Raw data should be presented in figures whenever informative to do so (typically when N per group is less than 10)
- For each experiment, you should identify the statistical tests used, exact values of N, definitions of center, methods of multiple test correction, and dispersion and precision measures (e.g., mean, median, SD, SEM, confidence intervals; and, for the major substantive results, a measure of effect size (e.g., Pearson's r, Cohen's d)
- Report exact p-values wherever possible alongside the summary statistics and 95% confidence intervals. These should be reported for all key questions and not only when the p-value is less than 0.05.

Please outline where this information can be found within the submission (e.g., sections or figure legends), or explain why this information doesn’t apply to your submission:

Page 11 (dataset 1) and page 13 (dataset 2)

(For large datasets, or papers with a very large number of statistical tests, you may upload a single table file with tests, Ns, etc., with reference to sections in the manuscript.)

**Group allocation**

- Indicate how samples were allocated into experimental groups (in the case of clinical studies, please specify allocation to treatment method); if randomization was used, please also state if restricted randomization was applied
- Indicate if masking was used during group allocation, data collection and/or data analysis

Please outline where this information can be found within the submission (e.g., sections or figure legends), or explain why this information doesn’t apply to your submission:

Groups (different non-learner definitions) were based on what had been done in the field previously as based on a systematic literature search

Figure 3, Figure 4

Supp Table 1 and Supp Table 2

**Additional data files (“source data”)**

- We encourage you to upload relevant additional data files, such as numerical data that are represented as a graph in a figure, or as a summary table
- Where provided, these should be in the most useful format, and they can be uploaded as “Source data” files linked to a main figure or table
- Include model definition files including the full list of parameters used
- Include code used for data analysis (e.g., R, MatLab)
- Avoid stating that data files are “available upon request”

Please indicate the figures or tables for which source data files have been provided:

Code is provided on OSF (see above) for all Figures that use experimental data – that is except for Figure 2 and Figure 5 which are based on the results of the systematic literature search (supp Tables 1 and 2)
